# Therapeutic potential of artemisinin and its derivatives in managing kidney diseases

**DOI:** 10.3389/fphar.2023.1097206

**Published:** 2023-02-15

**Authors:** Qi Jin, Tongtong Liu, Danqian Chen, Liping Yang, Huimin Mao, Fang Ma, Yuyang Wang, Ping Li, Yongli Zhan

**Affiliations:** ^1^ China Academy of Chinese Medical Sciences, Guang’anmen Hospital, Beijing, China; ^2^ China-Japan Friendship Hospital, Institute of Clinical Medical Sciences, Beijing, China

**Keywords:** artemisinin, qinghaosu (artemisinin), artesunate, dihydroartemisinin, artemether, kidney disease

## Abstract

Artemisinin, an antimalarial traditional Chinese herb, is isolated from *Artemisia annua. L*, and has shown fewer side effects. Several pieces of evidence have demonstrated that artemisinin and its derivatives exhibited therapeutic effects on diseases like malaria, cancer, immune disorders, and inflammatory diseases. Additionally, the antimalarial drugs demonstrated antioxidant and anti-inflammatory activities, regulating the immune system and autophagy and modulating glycolipid metabolism properties, suggesting an alternative for managing kidney disease. This review assessed the pharmacological activities of artemisinin. It summarized the critical outcomes and probable mechanism of artemisinins in treating kidney diseases, including inflammatory, oxidative stress, autophagy, mitochondrial homeostasis, endoplasmic reticulum stress, glycolipid metabolism, insulin resistance, diabetic nephropathy, lupus nephritis, membranous nephropathy, IgA nephropathy, and acute kidney injury, suggesting the therapeutic potential of artemisinin and its derivatives in managing kidney diseases, especially the podocyte-associated kidney diseases.

## 1 Introduction

Artemisinin, also known as *Qinghaosu*, is the main active ingredient of a traditional Chinese herbal medicinal plant *A. annua L*. It has demonstrated therapeutic potential in treating chronic and refractory diseases and may hold massive potential for developing modern drugs (Yao et al., 2022). The effectiveness and safety of artemisinin have been proven *via* several basic experiments and clinical trials. It has been used as a first-line drug for treating malarial, and artemisinin-based combination therapy has been widely used as a standard antimalarial therapy. Artemisinin is a hemiterpene lactone endoperoxide with several derivatives, including dihydroartemisinin (DHA), artesunate (ART), artemether, and arteether. These derivatives exhibited better efficacy and stability than artemisinin and were more helpful in treating malaria (Shi et al., 2022). Several pieces of evidence have shown that artemisinin and its derivatives benefit several other diseases like cancer, inflammatory diseases, and immune diseases (Chen et al., 2020; Cheong et al., 2020).

Podocyte injury occurs early in diabetic nephropathy (DN) (Jiang et al., 2022). However, in non-diabetic glomerular diseases, such as membranous nephropathy (MN), focal segmental glomerulosclerosis (FSGS), obesity-related glomerulopathy (ORG), and IgA nephropathy (IgN), abnormalities in the structure and function of podocytes were also observed (Jia et al., 2020; Alok and Yadav, 2022; Guruswamy Sangameswaran & Baradhi, 2022; Su et al., 2022). Substantial evidence suggests that proteinuria due to podocyte injury is the earliest event in glomerular diseases.

More specifically, podocytes have a limited ability to repair and regenerate, suggesting that most podocyte injury or loss is irreversible and is associated with the progression to end-stage renal disease (ESRD) in glomerular diseases (Zuo and Sun, 2021). Clinically,angiotensin-converting enzyme inhibitors and angiotensin II receptor blockers can prevent or reduce proteinuria and the occurrence of adverse events of glomerular disease, which can delay the progression of ESRD (Vaz de Castro et al., 2022). Understanding the pathogenesis and progression of proteinuric glomerular diseases, especially the podocytes, is essential. Therefore, developing drugs targeting renal podocyte injury and therapeutic strategies to repair podocyte injury remains challenging.

**FIGURE 1:**
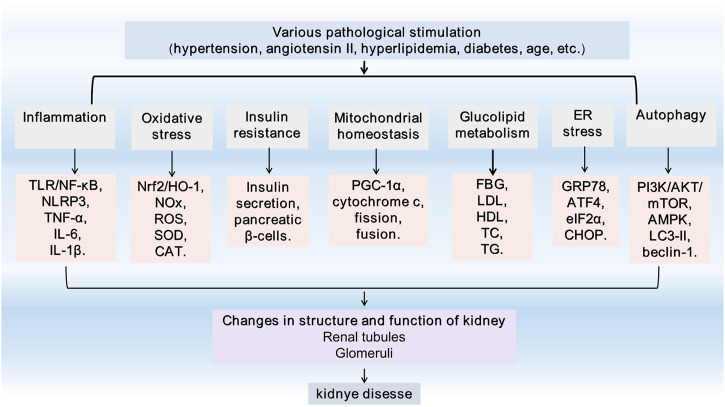
General feature of kidney injury. TLR4, Toll-like receptor 4; NF-κB, Nuclear factor-kappaB; NLRP3, Nucleotide leucine-rich polypeptide 3; IL-1, interleukin-1; TNF-α, tumor necrosis factor alpha; Nrf2, nuclear factor erythroid 2-related factor 2; HO-1, heme oxygenase-1; NADPH, Nicotinamide adenine dinucleotide phosphate; NOX, NADPH oxidase; ROS, reactive oxygen species; SOD, superoxide dismutase; CAT, catalase; PGC-1α, Peroxisome proliferator-activated receptor γ coactivator-1α; FBG, fasting blood glucose; TG,triglyceride; TC, total cholesterol; LDL, low-density lipoprotein; HDL, high-density lipoprotein; GRP78, glucose-regulated protein 78; ATF4, activating transcription factor 4; eIF2α, eukaryotic initiation factor-2α; CHOP, C/EBP homologous protein; PI3K, phosphatidylinositol 3-kinase; AKT, protein kinase B; mTOR, mammalian target of rapamycin; AMPK, adenosine monophosphate-activated protein kinase; LC3, microtubule-associated protein light chain 3; ER, endoplasmic reticulum.

There is much evidence (Xia et al., 2020a) that artemisinin and its derivatives have beneficial effects on kidney diseases, such as DN, lupus nephritis (LN), nephrotic syndrome (NS), acute kidney injury (AKI), chronic kidney diseases (CKD), IgAN (Figure 1). This study systematically reviewed the latest research progress in identifying the potential pathway of artemisinin and its derivatives involved in podocyte injury and related glomerular diseases for the clinical treatment of glomerular diseases and innovative strategies to delay and reverse kidney injury.

## 2 Pharmacological characteristics of artemisinin and its derivatives

**FIGURE 2:**
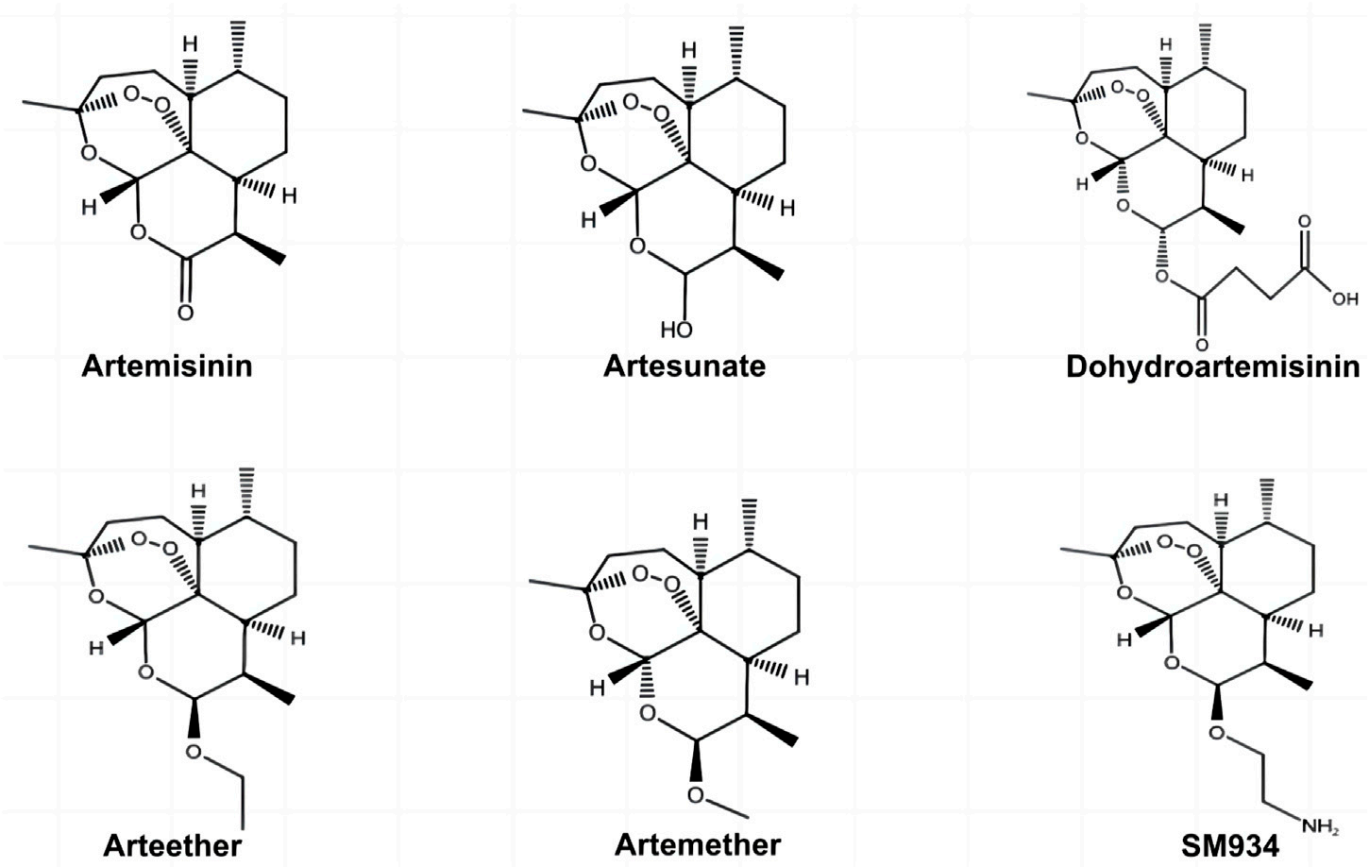
Chemical structures of artemisinin and its derivatives. SM934, *ß*-aminoarteether maleate.

A. annua L., a traditional Chinese herb, has therapeutic effects on several diseases. Inspired by Ge Hong’s *Zhouhou Beiji Fang* in the Eastern Jin Dynasty, artemisinin was extracted and isolated from A. annua L. by Youyou Tu and other scientists from the Chinese Academy of Chinese Medical Sciences. Its effectiveness and safety in basic experiments and clinical trials were confirmed. Artemisinin is a first-line drug for treating malaria, and artemisinin-based combination therapy has been widely used as the world’s standard antimalarial therapy. The discovery of artemisinin led Youyou Tu to win the 2015 Nobel Prize in Physiology or Medicine. Youyou Tu’s group focus on deducing the structure of artemisinin *via* mass spectrometry and other technical analysis. They demonstrated that artemisinin is a hemiterpene lactone endoperoxide without a nitrogen heterocyclic structure. It showed higher efficacy, lower toxicity, and fewer adverse effects than traditional antimalarial drugs, such as hydroxychloroquine and chloroquine (Talman et al., 2019; Ma et al., 2020). However, artemisinin exhibited poor water solubility and lipid solubility, poor stability, and low oral bioavailability, which limits its clinical applications. To improve artemisinin’s physical and chemical properties, researchers have modified and developed various derivatives of artemisinin, such as DHA, ART, artemether, arteether, and *ß*-aminoarteether maleate (SM934) (Figure 2). These derivatives are more commonly used to treat malaria than artemisinin due to their higher efficacy and stability (Shi et al., 2022). Several pieces of evidence have demonstrated the anti-cancer, anti-inflammatory, anti-viral, antioxidative stress, and anti-immune response of artemisinin and its derivatives (Tsui et al., 2019; Fu et al., 2020; Dolivo D and Dominko, 2021).

The pharmacokinetics studies of artemisinin and its derivatives have demonstrated fast absorption, wide distribution, fast excretion, and low *in vivo* concentration. The primary biologically active metabolite involved in the antimalarial activities of artemisinin and its derivatives is DHA (Aderibigbe, 2017). They have a fast onset of action. The half-life of artemisinin was 2–5 h, while that of ART and artemether was less than 1 h and 2–4 h, respectively. After oral administration, it can be absorbed by the gastrointestinal tract rapidly and is mainly distributed in organs of the liver, kidney, and bile. Approximately 80% of it is excreted through the urine and feces within 24 h after administration (Li, 2012). Dai et al. (2019) studied the distribution of artemisinin drugs in the blood of rats. The order of blood cell affinity was artemisinin > artemether > DHA, the area under the curve (AUC) 0∼t of artemisinin, artemether, and DHA in the blood cells were 2.6, 1.7, and 1.2 times than that in plasma, respectively. In addition, the absorption of artemisinin in the body has apparent gender and individual differences. After continuous oral administration of artemisinin in rats for five days, the AUC0∼t of females and males decreased by 63.5% and 56.4%, and the maximum plasma concentration (Cmax) decreased by 66.8% and 55.8% (Li et al., 2021).

Several clinical studies have demonstrated that artemisinin and its derivatives are safe, and no severe adverse reactions have been observed during the treatment. Common adverse effects include nausea, vomiting, diarrhea, transient transaminase elevation, and mild rash, which is mild and self-healing (Mssusa et al., 2016; Roussel et al., 2017).

Artemisinin-based combination antimalarial therapy still has high efficacy in uncomplicated falciparum malaria, and several coexisting diseases may affect the susceptibility of artemisinin to adverse drug reactions. The possibility of adverse drug reactions in patients with comorbidities was three times more than in those without comorbidities. The risk factors, including age, weight, and height, were associated with adverse events (Bassi et al., 2017). Several studies have shown that artemisinins have embryonic lethal and teratogenic effects; however, they have not been proven in humans; therefore, their use in the first three months of pregnancy is not recommended (Gomes et al., 2016). In conclusion, the safety of artemisinin drugs is very high, but its adverse reactions cannot be ignored, especially when long-term medication is required. Therefore, it is recommended that clinicians pay close attention to patients’ reactions after taking the drugs to prevent or minimize adverse reactions (Amorim et al., 2013).

## 3 Role of artemisinin and its derivatives in kidney disease

Several studies have demonstrated that many important traditional Chinese medicines, extracts, and their active ingredients regulate inflammation, oxidative stress, autophagy, mitochondrial homeostasis, endoplasmic reticulum (ER) stress, glucose, and lipid metabolism, which reduces the injury caused by kidney-induced effects in response to various pathological stimuli (Tang et al., 2021; Wongmekiat et al., 2021; He et al., 2022; Jin et al., 2022; Wu et al., 2022). According to the existing literature, artemisinin and its derivatives have a particular regulatory effect on animal models of kidney diseases (Table 1).

**TABLE 1:** Effect of artemisinin and its derivatives on various kidney diseases.

Diseases	Types of artemisinin	Dosage	Effect↓	Effect↑	References
DN	artemether	0.67 g/kg/d, 12w	UAE, GBM, FPW, TBM, KW, HbA1c, UGE	PGC-1α, MPC, insulin	Han et al. (2019)
	artemether	0.67 g/kg/d, 8w	PDK1, UAE, NAG, NGAL, Kim-1, KW, HbA1c,FBG, UGE	PGC-1α, CAT, TP, ALB, insulin	Wang et al. (2019c)
	artemisinin	300 mg/kg/d,4w	FBG, UAE, BUN, Cr, LDL-C, TG, TC	insulin	Xiang et al. (2019)
	artemisinin	25,50,75 mg/kg/d,8w	24 h UAER, BUN, GBM, Scr, MDA, TGF-β1, KI, FBG	BW, T-SOD, GSH-Px, Nrf2, HO-1, NQO1	Zhang and Song (2020)
	artemether	T1DM:1.6 g/kg/d,8w; T2DM:0.67 g/kg/d,12w	FBG, UAE, NAG, HbA1c, KI, KI/BW, UGE	ALB, TP	Rong et al. (2022)
	artemether	0.8 g/kg/d,8w	UAE, UACR, FBG, KW	CAT, ALB, TP, SOD	Chen et al. (2022)
LN	ART	125 mg/kg/d,16w	ANA, SCr, MCP-1,anti-dsDNA antibodies, BAFF, proteinuria	BW	Jin et al. (2009)
	ART	5.10 mg/kg/d,8w	IL-6, IFN-γ, IL-21, Tfh,a nti-dsDNA antibodies	Tfr/Tfh	Dang et al. (2019)
	SM934	10 mg/kg/d,21d	BUN, UACR, ANA, IL-1β, IL-6, TNF-α, IL-17, IL-23, anti-CL, anti-dsDNA IgG	Nrf2,HO-1	Lin et al. (2021)
	artemisinin	150 mg/kg/d, 8w	24 h urine protein, TNF-α, IL-6, NF-kBp65, NF-kB, TGF-β1		Wu et al. (2010)
	artemisinin	5.55 mg/kg/d,8w	UAE, SCr,anti-dsDNA antibodies, ANA, IgG, NF-κB, IFN-γ, TNF-α, TGF-β1	KLF15	Liang et al. (2018)
	SM934	2.5,5,10 mg/kg/d,18w	Proteinuria, BUN, IgG,IL-6, IL-10, IL-21, ANA, TLR7, TLR9, MyD88, anti-dsDNA IgG, anti-dsDNA IgM	ALB	Wu et al. (2016)
	artemisinin	150 mg/kg/d,8w	24 h urine protein, GRβ mRNA	GRα mRNA, transcriptional coactivator P300/CBP protein	Wu et al. (2011)
MN	Artemisinin	12.5.25 mg/kg/d,4w	proteinuria, IgG,desmin,KW/BW, C3, C5b-9, ECM, α-SMA, TGF-β1, p-Smad2, p-Smad3	ALB, podocin, nephrin	Li et al. (2015)
IgAN	artemisinin	33.33 mg/kg/d,16w	24 h urine protein, UREA, TG, IgA, IgG, C3, xp-p65, IκB-α,NLRP3, ASC, caspase-1, IL-1β	TP, exosomes protein: CD63, CD81	Bai et al. (2019b)
	artemisinin	33.33 mg/kg/d,16w	IgA, C3, IL-4, IL-17, Th2/Th17	IFN-γ,Th1/Treg	Bai et al. (2019a)
AKI	ART	7.5.15 mg/kg/d,3d	Scr, BUN, Tim-1, IL-1β, IL-6, TNF-α, NO, NF-κB		Lei et al. (2021)
	DHA	20 mg/kg/d,3d	Scr, BUN, caspase-3, IL-1β, IL-5, IL-6, IL-17A, IFN-γ, TNF-α, CXCL1, MCP-1, MIP-2, p-p65, p-IκBα, MDA, NO	BW, GSH, CAT, SOD	Liu et al. (2019)
	DHA	50 mg/kg/d,1d	UACR, SCr, TNF-α	occludin	Cheng et al. (2018)

Abbreviation: DN, diabetic nephropathy; LN, lupus nephritis; MN, membranous nephropathy; IgN, immunoglobulin A nephropathy; AKI, acute kidney injury; DHA, dihydroartemisinin; ART, artesunate; SM934, *ß*-aminoarteether maleate; UAE, urinary albumin excretion; GBM, glomerular basement membrane; TBM, tubular basement membrane; FPW, foot process width; KW, kidney weight; UGE, urinary glucose excretion; BUN, blood urea nitrogen; SCr, serum creatinine; UAER, urinary albumin excretion rate; UACR, urine albumin to creatinine ratio; Kim-1, kidney injury molecule-1; PDK1, pyruvate dehydrogenase kinase 1; NAG, N-acetyl-β-d-glucosaminidase; NGAL, neutrophil gelatinase-associated lipocalin; KI, kidney index; BW, body weight; Tim-1, tubular injury molecule-1; HbA1c, hemoglobin A1c; FBG, fasting blood glucose; IL-6, interleukin-6; TNF-α, tumor necrosis factor-α; MCP-1, monocyte chemoattractant protein-1; NF-κB, nuclear factor-κB; TLR, Toll like receptor; MyD88, Myeloid differentiation primary response protein 88; IκB-α, inhibitors of κB-α; NLRP3, nucleotide-binding domain and leucine-rich repeat protein 3; MIP-2, macrophage inflammatory protein-2; TGF-β1, transforming growth factor β1; IFN-γ, interferon γ; ANA, antinuclear antibody; anti-dsDNA, anti-double-stranded DNA; IgG, immunoglobulin G; C3, complement 3; BAFF, B cell activating factor; Th, T helper cells; Treg, T regulatory cells; Tfh, follicular helper T cells; Tfr, follicular regulatory T cells; GR, GC, receptor; α-SMA, α-smooth muscle actin; P-Smad, Phosphorylated Smad; TC, total cholesterol; LDL-C, low-density lipoprotein cholesterol; TG, triglycerides; TP, total protein; ALB, albumin; NO, nitric oxide; MDA, malondialdehyde; CAT, catalase; SOD, superoxide dismutase; T-SOD, total superoxide dismutase; GSH, glutathione; GSH-Px, glutathione peroxidase; NQO1, benzoquinone reductase; CXCL1, chemokine (C-X-C motif) ligand 1; PGC-1α, peroxisome proliferator-activated receptor-γ co-activator-1α; MPC, mitochondrial pyruvate carrier; Nrf2, nuclear factor E2 related-factor 2; HO-1, hemeoxxygenase-1; KLF15, Kruppel-like factor 15.

### 3.1 Inflammation

**FIGURE 3:**
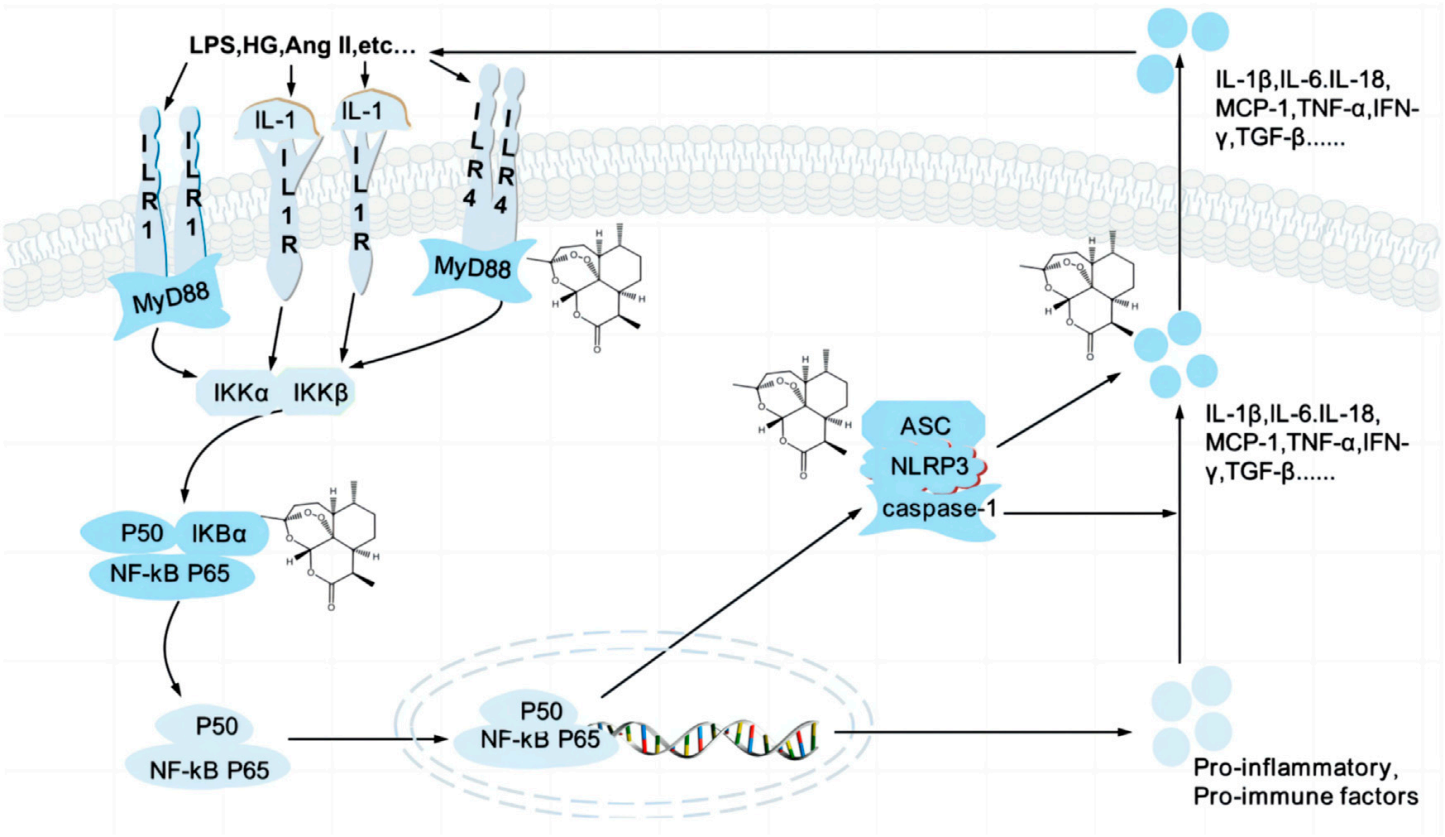
Artemisinin and its derivatives target inflammation to regulate kidney injury. High glucose, AngⅡ. Trigger activation of TLR/NF-κB signaling pathway and NLRP3 inflammasome, release inflammatory cytokines and chemokines and induce inflammatory responses, which lead to podocyte injury. Artemisinin and its derivatives can regulate various targets of the signaling pathway (structural formula of artemisinin), inhibit the formation of inflammatory factors and reduce the secretion of inflammatory factors, reduce the inflammatory response, and repair kidney injury. TLR4, Toll-like receptor 4; MyD88, myeloid differentiation factor 88; LPS, lipopolysaccharide; HG, high glucose; Ang II, angiotensin II; TNF-α, tumor necrosis factor alpha; IL-1, interleukin-1; IKK, an inhibitor of kappa B kinase; NF-κB, Nuclear factor-kappaB; NLRP3, nucleotide leucine-rich polypeptide 3; MCP, monocyte Chemoattractant Protein; IFN-γ, interferon-gamma; TGF-β, transforming growth factor *ß*.

The podocytes are the primary target cells for abnormal inflammation and immunity in various kidney diseases. Several studies demonstrated the benefits of immunosuppressive therapy for podocyte injury-associated kidney diseases such as FSGS and MN(van de Logt et al., 2019; Trachtman, 2020), effectively reducing proteinuria and kidney injury in DN patients (de Zeeuw et al., 2015) (Figure 3).

#### 3.1.1 TLR/NF-κB pathway

Nuclear factor-kappaB (NF-κB) is a transcription factor regulating the expression of the κ light chain, including p50, RelA/p65, and IκBα, which further restrict the expression of inflammatory factors and chemokines. NF-κB is usually present in the cytoplasm, and in response to various pathological stimuli, IκBα is phosphorylated, NF-κB is released and translocated into the nucleus, thereby targeting the activation of a series of factors, including tumor necrosis factor-alpha (TNF-α), interleukin-1β (IL-1β), and IL-6. The receptor activator of nuclear factor-kappaB (RANK), a type I membrane protein, is a crucial activator of NF-kB. High glucose (HG) and nicotinamide adenine dinucleotide phosphate (NADPH) oxidase (NOX) 4 induced the overexpression of RANK in the podocytes, P22phox was increased *via* p65, and the production of pro-inflammatory factors was induced, such as TNF-α and IL-1β, was generated, leading to podocyte injury (Ke et al., 2021). NF-κB inhibitors were used in a model of podocyte injury model, the activation and phosphorylation of IκKα, IκBα, and NF-κB p65 were inhibited, the cytokines levels of IL-6, TNF-α, monocyte chemoattractant protein-1 (MCP-1) and transforming growth factor *ß* (TGF-β) 1 were decreased, which could effectively repair podocyte injury, reduce proteinuria and relieve edema (Wang et al., 2018; Zhu et al., 2019).

Toll-like receptor 4 (TLR4) is a family of innate immune recognition receptors which can activate myeloid differentiation factor 88 (MyD88) and NF-κB. A study by Ren F’s demonstrated that psoriasis-like inflammation activated TLR receptors, upregulated the expression of TLR2 and TLR4, activated MyD88 receptor and NF-κB pathway, increased the expression of NF-κB p65, IL-1β, IL-6, TNF-α, and other factors, causing podocytes injury (Ren et al., 2020). Conversely, inhibition of TLR4/NF-κB signaling can slow podocyte injury of DN (Yang et al., 2017).

Interestingly, artemisinin and its derivatives have potent anti-inflammatory and anti-immune. Zhao X demonstrated that artemisinin modulated the TLR4/NF-κB signaling and delayed microglia’s activation of immune-inflammatory responses by microglia (Zhao et al., 2022). Chen et al. (2021b) exhibited that ART reduced the expression of TLR-4, MyD88, NF-κB, TNF-α, and IL-6 in ischemic cortical regions through the TLR-4/NF-κB pathway. Chen YX confirmed that ART could reduce the expression levels of TLR4, p-NF-κB, p-p38, Bcl2 associated X (Bax), and caspase-9, increase the expression of B cell lymphoma-2 (Bcl-2) in a dose-dependent manner, inhibit the TLR4/NF-κB signaling pathway, and downregulate the levels of TNF-α, IL-8 and interferon-gamma (IFN-γ) (Chen et al., 2019). To further explore the role of ART in kidney diseases, it was observed that ART inhibited the protein expression of α-smooth muscle actin (α-SMA), TLR4, MyD88, NF-κB p65, and TGF-β1, and decreased the levels of TNF-α, IL-6, and caspase-3, which can inhibit the inflammation in mice with nephritis through the TLR4/NF-κB signaling pathway (Wan and Li, 2017).

SM934 inhibited TLR-triggered B cell activation and proliferation, as well as antibody secretion, decreased serum cytokines of anti-nuclear antibodies, IL-6, IL-10, and IL-21 by downregulating the TLR7/9 mRNA expression, and protected MRL/LPR mice from LN (Diao et al., 2022). Furthermore, DHA also inhibited the elevation in p-IKKα/IκBα and NF-κB p65 *in vivo* and *in vitro*, meanwhile inhibiting the production of pro-inflammatory cytokines, including TNF-α, IL-6, and IL-1 (Li and zhou, 2019) significantly reduced the TLR4 level and the activation of MyD88, IRAK4, and NF-κB, and delayed the progression of LN (Diao et al., 2019).

#### 3.1.2 NLRP3 inflammasomes

Nucleotide leucine-rich polypeptide 3 (NLRP3) inflammasomes are an essential member of the nucleotide-binding oligomerization domain-like receptor family, composed of NLRP3, apoptosis-related spot-like protein (ASC) and caspase-1, which can recognize pathogenic microorganisms or cell damage signals, initiate innate immune responses, and promote inflammatory responses. An in-depth study of the inflammasomes showed that the dysregulation of NLRP3 inflammasomes activation is closely related to various kidney diseases, including chronic glomerulonephritis, IgAN, and LN (Fu et al., 2017). The podocytes contain large amounts of the NLRP3 inflammasomes (Kim et al., 2018), stimulated by HG and lipopolysaccharide (LPS), the NLRP3 inflammasomes in podocytes are activated to promote the activation and secretion of IL-1β, IL-8, IL-18, IL-18, and caspase-1 (Kim et al., 2019; Ying et al., 2022). The activation of NLRP3 inflammasomes also leads to the cytoskeleton rearrangement of podocytes (Zhang et al., 2012). Inhibition of NLRP3 inflammasome activation could attenuate podocyte injury and improve renal function in kidney diseases caused by various factors (Wu et al., 2018), such as inflammation and lipid metabolism in DN (Wu et al., 2021).

Artemisinin can reduce the activation of the NLRP3 inflammasomes, such as downregulating the levels of NLRP3, ASC, caspase-1, and IL-1β(Wang et al., 2020). NF-κB is one of the upstream molecules of NLRP3. The pretreatment with artemisinin could significantly prevent the activation of NF-κB and NLRP3 inflammasomes in angiotensin II (Ang II)-induced injury of the human kidney 2 (HK-2) cells. At the same time, BAY11 -7082 (NF-κB inhibitor) significantly inhibited the activation of NLRP3 inflammasomes (Wen et al., 2019). Moreover, DHA also suppressed the formation of NLRP3 inflammasomes (Liang et al., 2020). As a water-soluble artemisinin derivative, SM934 reduced the accumulation of TLR4, inhibited the expression of inflammatory components, including MyD88, NLRP3, ASC, and caspase-1, and also reduced the inflammatory mediators (TNF-α, IL-6, IL-10 or IL-1β) by regulating TLR4/NF-κB/NLRP3 signaling (Yang et al., 2021). Sun Z evaluated ART’s effects and molecular mechanisms on the DN in an *in vitro* model. ART reduced the expression levels of TLR4, MyD88, p-p65NF-κB, and NLRP3 induced by HG and decreased inflammatory cytokines, such as IL-6, IL-1β, and TNF-α inhibited the TLR4/NF-κB pathway and the expression of NLRP3 inflammasomes (Sun et al., 2018). ART combined with hydroxychloroquine inhibited the expression of NF-κB and NLRP3 inflammasomes, including IκB-α, p-p65, NLRP3, ASC, IL-1β, and caspase-1, which reduced the twenty-four-hour urinary protein, immune complex deposition levels of IgA and IgG in IgAN rats, and the effects were consistent *in vitro* and *in vivo* (Bai et al., 2019b).

### 3.2 Oxidative stress

Oxidative stress is caused by an imbalance between oxidant production and antioxidant reserves and is responsible for the onset and progression of various kidney diseases. Podocytes are susceptible to oxidative stress, which lead to podocyte dysfunction and proteinuria. In puromycin aminonucleoside (PAN)-induced minimal change disease (MCD), aldosterone/Ang II-Rac1-mediated hypertensive nephropathy and DN, etc., podocytes generate reactive oxygen species (ROS), which attacks normal cells and leads to renal dysfunction (Nagata, 2016). Extracellular superoxide dismutase (SOD), an antioxidant, is expressed at high levels in normal adult kidneys compared to the wild-type controls. Mice that were knocked out for extracellular SOD were susceptible to doxorubicin and chronic Ang II, induced glomerular injury, and developed more severe proteinuria (Tan et al., 2015).

#### 3.2.1 Nrf2/HO-1 signaling pathway

The nuclear factor erythroid 2-related factor 2 (Nrf2)/heme oxygenase-1 (HO-1) signaling pathway is a classic antioxidant pathway. Nrf2 and Kelch-like ECH-associated protein-1 (Keap1) form the Nrf2-Keap1 complex and are stably present in the cytoplasm physiologically. When excessive ROS is produced, Nrf2 changes from a bound state to a free state, and moves to the nucleus, where it binds to the antioxidative response element (ARE) in the promoter region to induce the transcription of antioxidant enzymes, such as SOD and glutathione-S-transferases, which further improves the scavenging ability of ROS. HO-1 is a target gene of Nrf2, which can accelerate the degradation of heme to generate biliverdin, carbon monoxide, and ferrous iron (Loboda et al., 2016), and is involved in regulating podocytes (Facchinetti, 2020). Induction of the Nrf2/HO-1 signaling significantly reduced podocyte loss, immune complex deposition, and proteinuria in a PHN model (Wang et al., 2021). In an Ang II-induced podocyte injury model, Sirt6 protected podocytes by enhancing the activation and expression of Nrf2 and HO-1 genes in the podocytes (Fan et al., 2021). The Nrf2/HO-1 signaling pathway activation could reduce FP fusion of podocytes, upregulate synaptopodin, downregulate desmin, repair podocyte injury, and improve proteinuria in the MCD rat model (Wang et al., 2019b). After knocking out Nrf2, ROS was significantly increased, compared with the standard control group, and the expressions of Nrf2 and HO-1 proteins in the mouse podocyte clone (MPC5) cells induced by HG were significantly decreased. After treatment, the expression of Nrf2 and HO-1 proteins were significantly increased and accompanied by a reduction in ROS (Zhao et al., 2019). In addition, the activation of the Nrf2/HO-1 axis can effectively inhibit the activation of the NLRP3 inflammasomes and protect podocytes from inflammatory injury in DN (Ding and Qiao, 2022).

Artemether is a lipid-soluble peroxide sesquiterpenoid compound used clinically as an antimalarial drug. In addition, it has potent antioxidant activity, and its effects indirectly stimulate the endogenous antioxidant system in the body. Artemether-induced phosphorylation of the adenosine monophosphate-activated protein kinase (AMPK)/glycogen synthase kinase 3β (GSK3β) pathway, which activated the Nrf-2/AREs1 protein, increased the levels of HO-1 and SOD and decreased the production of ROS(Li et al., 2019), and attenuated kidney injury in type 1 diabetic and db/db mice (Wang et al., 2019c; Han et al., 2019). ART and DHA can also activate Nrf2 (Li et al., 2019a), inhibit ROS-induced apoptosis and caspase-3 activity, and reduce the ratio of BAX/Bcl-2 (Lu et al., 2018). The DN rat model was established by intraperitoneal injection of streptozotocin (STZ); artemisinin inhibited the expression of TGF-β1 protein in renal tissues, activated the Nrf2 signaling pathway, enhanced the expression of antioxidant proteins, and significantly increased the activity of T-SOD and glutathione peroxidase (GSH-Px), reduced malondialdehyde (MDA), alleviated the oxidative stress injury in the early kidney of DN rats (Zhang and song, 2020). Further studies demonstrated that artemisinin activated Nrf2 by increasing its stability and reducing ubiquitination (Liu et al., 2019).

#### 3.2.2 NOX

As a source of ROS, the NOX family consists of seven isoforms (NOX1-5 and Duox1-2). Several studies have shown that the overexpression of NOX1, NOX4, and NOX5 can cause podocyte injury and promote the onset and development of DN (Shao et al., 2020). HG can activate NOX by advanced glycation end products (AGEs)/receptor of advanced glycation end products (RAGE) (Pathomthongtaweechai N, 2020). In addition, it can directly mediate the translation of NOX4 and promote the production of ROS (Shi et al., 2020). Advanced oxidation protein products (AOPPs)-induced NOX activation *via* RAGE and stimulated the production of ROS and NF-κB. In contrast, the levels of nephrin, podocin, and zona occludens 1 (ZO-1) were decreased, and the expression of desmin and fibronectin in the podocytes increased. The podocyte phenotype changed, which caused a barrier to filtration and proteinuria (Zhou et al., 2019). Diabetes mellitus promotes NOX4 expression in the podocytes and the production of ROS, leads to the loss of podocytes, and damages glomeruli, which indicates that NOX4 is an attractive therapeutic target for DN (Rajaram et al., 2019).

Systematic review and meta-analysis showed that artemisinin has a protective effect on DN (Feng et al., 2022). STZ-induced experimental DN model was treated with artemether combined with enalapril; the expression of catalase (CAT) was upregulated, SOD and urinary albumin excretion (UAE) were downregulated, and glomerular hypertrophy was relieved (Chen et al., 2022). Spontaneously hypertensive rats were intervented with long-term artemisinin, the endothelial nitric oxide synthase (eNOS) was increased, and the expression of NOX-2 and NOX-4 was decreased (Liu et al., 2021b). DHA upregulated total antioxidant capacity levels, glutathione reductase (GR), GSH-Px, and CAT, while downregulating pro-oxidative substances, including hydrogen peroxide, total NOS, and inducible NOS (Xiong et al., 2022). DHA reduced the levels of MDA, and nitric oxide in LPS-induced AKI, increased the levels of GSH, CAT, and SOD, restored balance between oxidative and antioxidant and ameliorated LPS-induced renal oxidative stress, which may contribute to the protection of DHA against LPS-induced AKI (Liu et al., 2019). ART also significantly reversed the expression and activity of SOD and CAT (Ho et al., 2012).

### 3.3 Autophagy

**FIGURE 4:**
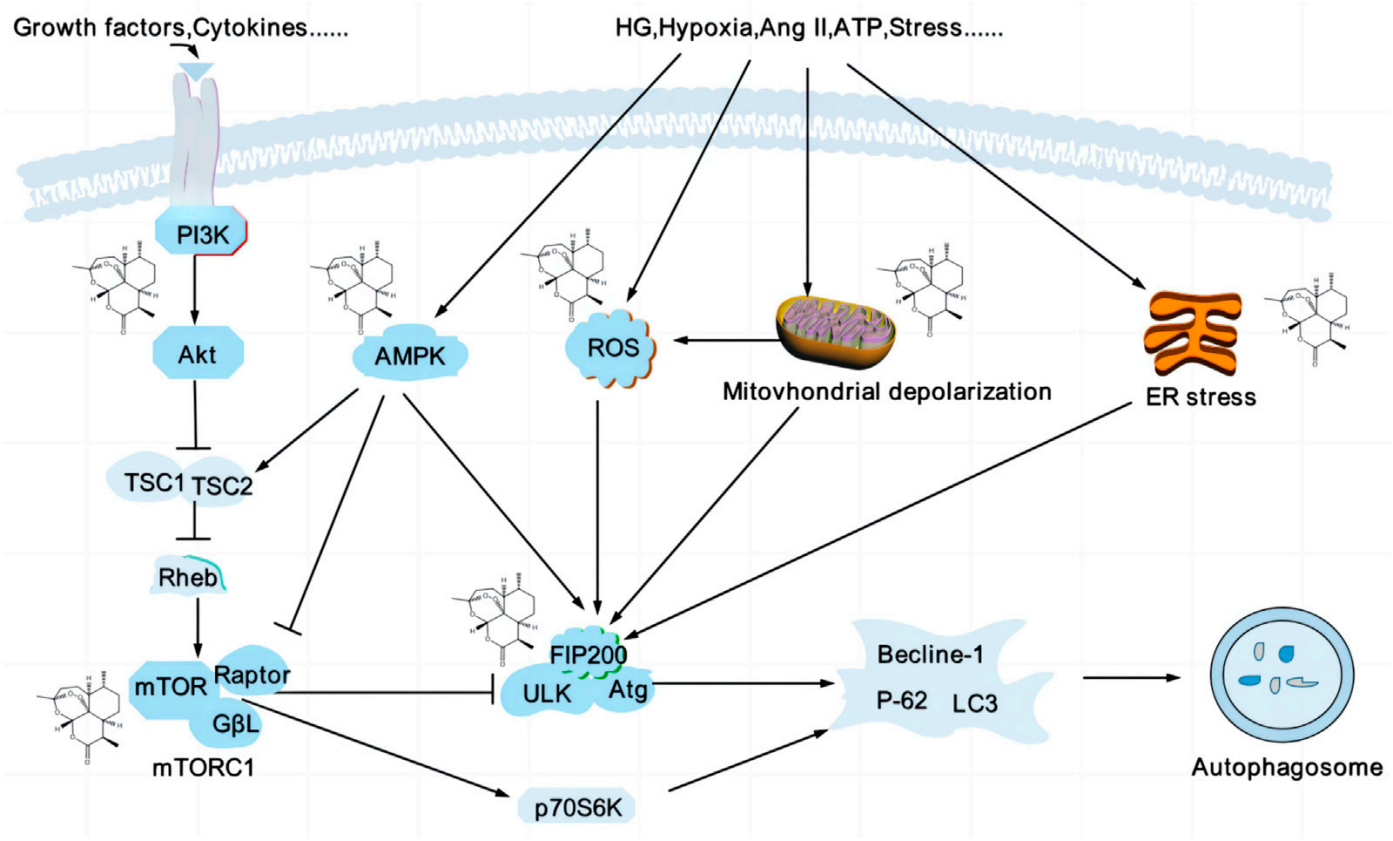
Artemisinin and its derivatives modulate autophagy to delay kidney injury. Podocyte autophagy is affected by various factors, such as inflammatory factors, oxidative stress, mitochondrial depolarization, and endoplasmic reticulum stress, etc. mTOR and AMPK are significant regulators of autophagy, and mTORC1 is a negative regulators of autophagy, inhibiting autophagy by regulating the ULK1 complex. AMPK can promote autophagy by inhibiting mTORC1 or directly phosphorylating the expression of several autophagy-related proteins, such as ULK1, Atg, and beclin1. Artemisinin and its derivatives regulate related targets (structural formula of artemisinin) and regulate the process of autophagy. PI3K, phosphatidylinositol 3-kinase; AKT, protein kinase B; mTOR, mammalian target of rapamycin; AMPK, adenosine monophosphate-activated protein kinase; ULK, unc-51-like kinase; Atg, autophagy-related genes; ROS, reactive oxygen species; HG, high glucose; Ang II, angiotensin II; ATP, adenosine triphosphate; ER, endoplasmic reticulum; LC3, microtubule-associated protein light chain 3.

Podocytes are highly differentiated cells lacking in regenerative capacity, which cannot be repaired by cell proliferation after injury, leading to an irreversible glomerular injury. Therefore, the autophagy-lysosome system is crucial for maintaining podocyte homeostasis. Under normal conditions, glomerular podocytes have a high level of basal autophagic activity to remove and degrade unfolded/misfolded proteins and senescent/damaged organelles to maintain a stable intracellular environment (Choi, 2020). Autophagy is regulated by various autophagy-related proteins and autophagy pathway protein complexes, such as autophagy-related genes 5 (Atg5), microtubule-associated protein light chain 3 (LC3)-II, beclin-1, uncoordinated 51-like kinase (ULK)1/Atg13/focal adhesion kinase family interacting protein of 200 kDa (FIP200) complex, vacuole protein sorting 34/phosphatidylinositol 3-kinase (Vps34)/phosphatidylinositol 3-kinase (PI3K) complex, Atg5/Atg12 coupling system (Bork et al., 2020), and the ULK1/Atg13/FIP200 complex are necessary for triggering autophagy (Vucicevic et al., 2020), the ratio of LC3-II/LC3-I as an indicator of autophagic flux. Podocyte autophagy disorders are associated with the development of various glomerular diseases, including FSGS and DN (Lu et al., 2021). The autophagy inhibitor 3-methyladenine (3-MA) reduces autophagosome formation, reduces autophagic function, and leads to the elevation of blood urea nitrogen (BUN) and creatinine (Cr), which autophagy activators can reverse (Guan et al., 2015); the depletion of Atg5 in DN could cause the defective autophagy and aggravate proteinuria and glomerulopathy (Koch et al., 2020). Various pathways can modulate autophagy in the kidney; the PI3K/protein kinase B (AKT)/mammalian target of rapamycin (mTOR) and AMPK play critical roles in regulating autophagy flux (Packer, 2020) (Figure 4).

#### 3.3.1 PI3K/AKT/mTOR pathway

The PI3K/AKT/mTOR pathway is one of the mechanisms of regulating autophagy in the podocytes. After external stimuli phosphorylated PI3K, AKT is activated by phosphorylation, and then mTOR is phosphorylated, which influences the transcription and translation of Atg by activating downstream effectors (Liu et al., 2015). The activation of the PI3K/AKT/mTOR signaling pathway leads to abnormal structure and function of glomerular podocytes, the decreased synthesis of ZO-1 and nephrin, the adhesion effects of podocytes to the glomerular basement membrane were decreased, podocyte shedding, and disruption of the integrity of the SD. The intervention of the PI3K/AKT/mTOR signaling pathway and autophagy axis may become a new target for treating kidney diseases caused by podocyte injury (Wang et al., 2019d; Tu et al., 2019). mTOR, including mTORC1 and mTORC2, is a negative regulator of podocyte autophagy. In MPC5 cells and DN rats with stimulation of HG, the mTOR pathway was activated, p62 was accumulated, the ratio of LC3II/LC3I was downregulated, and the autophagy flux was decreased (Zhang et al., 2021b). As an inhibitor of mTOR, rapamycin could reverse this phenomenon (Jin et al., 2022).

As a derivative of artemisinin, DHA can enhance the autophagy proteins, including beclin-1, and LC3-II, inhibiting the PI3K/AKT/mTOR pathway (Zhang et al., 2022a). Liu J demonstrated that DHA increased the LC3-II and the number of green fluorescent protein (GFP)-LC3 puncta, induced autophagy by inhibition of the Akt/mTOR pathway (Liu et al., 2019). In addition, DHA can downregulate mTOR/ribosomal protein S6 kinase *ß*-1 signaling pathway targets, promote autophagy, and improve IgAN mesangial cell proliferation (Xia et al., 2020b). ART decreased the expression of PI3K, AKT, mTOR, p-PI3K, p-AKT, p-mTOR, Bcl-2, and Bcl-xl and increased the expression of Bax, LC3II/LC3I, and beclin-1, which accelerated apoptosis and autophagy by inhibiting the PI3K/AKT/mTOR signaling pathway (Feng, 2018). Wan Q also confirmed that ART induced beclin-1-mediated autophagic flux *via* the PI3K/AKT/mTOR pathway. Interestingly, the genetic deletion of beclin-1 abolished ART-triggered autophagy (Wan et al., 2019).

#### 3.3.2 Activated protein kinase pathway

AMPK is a positive regulator of autophagy and an upstream regulator of mTOR. In ischemia and hypoxia cases, activated AMPK directly inhibits mTOR by phosphorylating Raptor, a subunit of mTORC1, and indirectly inhibits mTOR, enhancing mTOR autophagy by inhibiting the Rheb activity (Liu et al., 2021a). The latest study showed that AMPK and ULK1 primarily controlled autophagy in the podocytes. With the addition of AICAR, an AMPK agonist in podocyte-specific knockout Raptor mice, high levels of autophagy were observed, and the effects were durable and independent (Bork et al., 2020).

In various studies, artemisinin and its derivatives induced autophagy (Guan and Guan, 2020; Chen et al., 2021a), Cao Q found that artemisinin promoted AMPK activation, inhibited phosphorylation of mTOR and ULK1, increased LC-3II accumulation, and P62 degradation, and enhanced macrophage autophagy (Cao et al., 2020). Zhao F proved that ART induced autophagy by increasing the ratio of LC3B-II/LC3B-I (Zhao et al., 2020), which was consistent with Li L’s study, ART upregulated the expression of beclin-1 and the ratio of LC3II/I, downregulated p62, which enhanced autophagy in diabetic rats, meanwhile, the AMPK/SIRT1 pathway was also activated (Li et al., 2021a). Furthermore, ART triggers the upregulation of ROS, which initiates the AMPK-mTOR-ULK1 axis to induce autophagy-dependent apoptosis (Zhou et al., 2020).

### 3.4 Mitochondrial homeostasis

Various kidney cells require sufficient energy to maintain their normal functions; therefore, mitochondrial homeostasis plays a significant role in the normal biological processes of podocytes. Multiple factors lead to disturbance in mitochondrial adenosine triphosphate production, oxidative stress, inflammatory response, mitochondrial dynamics, mitophagy, and biosynthesis disturbance, resulting in disruption of energy metabolism of podocyte and podocyte injury (Gujarati et al., 2020). Growing research focused on the role of mitochondrial homeostasis in podocyte injury (Audzeyenka et al., 2022), and mitochondrial dysfunction is a key driver in the pathogenesis of podocyte injury and podocyte injury-associated kidney diseases, such as DN, CKD, and FSGS, (Wei and Szeto, 2019). As a critical regulator of mitochondrial biosynthesis, Peroxisome proliferator-activated receptor γ coactivator-1α (PGC-1α) can regulate oxidative phosphorylation and mitochondrial anaerobic glycolysis, and mice with specific knockout of PGC-1α showed dysfunction mitochondrial function (Svensson et al., 2016). Under mild oxidative stress or a small amount of ROS, PGC-1α can be effectively activated by regulating mitochondrial biosynthesis and reducing mitochondrial oxidative stress. In the *in vitro* experiments of the human podocytes, the levels of PGG-1α were significantly decreased under the induction of HG (Imasawa et al., 2017), accompanied by reduced mitochondrial and mitochondrial membrane potential, and increased ROS levels (Fan et al., 2019).

Artemisinin can significantly restore nuclear morphology, attenuate the accumulation of ROS in a concentration-dependent manner, reduce activation of mitochondrial membrane potential and caspase-3, and alleviate cell apoptosis (Zhao et al., 2019b). Qin YR proved that ART could reduce mitochondrial ROS production, delay mitochondrial fission, restore mitochondrial fusion and fission kinetics, promote autophagy and mitochondrial biosynthesis, and maintain mitochondrial homeostasis (Qin et al., 2022). Artemether regulated mitochondrial pyruvate metabolism, increased mitochondrial biosynthesis, and improved mitochondrial function (Cheng et al., 2022). Artemether significantly increased the rate of mitochondrial hydrogen peroxide release and upregulated the expression of CAT. It increased the levels of PGC-1α while decreasing the levels of the pyruvate dehydrogenase kinase 1 of mitochondria, modulating the redox balance in the kidney, and reducing proteinuria in STZ-induced DN (Wang et al., 2019c). Artemether increased voltage-dependent mitochondrial anion channel, translocase of the outer mitochondrial membrane 20, cytochrome c oxidase IV, mitochondrial transcription factor, and improved doxorubicin-induced renal injury (Han et al., 2021).

### 3.5 Endoplasmic reticulum stress

Endoplasmic reticulum (ER) stress is an adaptive response of cells. When cells are subjected to external stimuli, the ER will strengthen the cells against harmful stimuli by increasing the degradation and transport of proteins. However, when the strength of external stimulation exceeds the homeostasis of ER, it leads to unfolded protein response (UPR) and ER-associated protein degradation (ERAD) in ER (Zhang et al., 2020). There are abundant glycoproteins outside the podocyte membrane, ER is essential for maintaining protein homeostasis, and ER stress has a critical effect on DN and other kidney diseases (Cao et al., 2016). In models of kidney disease, ER stress was significantly increased, accompanied by ultrastructural changes in the ER of podocytes, including ER expansion, manifested by impaired glycosylation and decreased nephrin expression, and proteinuria gradually appears with age (Cybulsky, 2017). PAN-induced nephropathy model mice and podocytes, ER stress, was activated, and podocytes’ cytoskeletal proteins were disrupted, including synaptopodin, nephrin, and podocin (Long et al., 2022). ER stress was triggered by Ang II in podocytes of CKD, manifested by elevated levels of ER stress-related proteins, including glucose-regulated protein 78, activating transcription factor 4 (ATF4), eukaryotic initiation factor-2α (eIF2α), and C/EBP homologous protein (CHOP) (Yu et al., 2020). Tauroursodeoxycholic acid, a specific inhibitor of ER stress, was used in db/db mice, and the expression of RTN1A (a novel ER stress marker) in podocyte was decreased, the activation of ER stress was inhibited, podocyte injury and proteinuria in DN mice were ameliorated (Fan et al., 2017).

ART could inhibit ER stress and prevent ER stress-related apoptosis in tissues by preventing the activation of PERK-eIF2α-ATF4-CHOP and IRE1α-XBP1 signaling pathways (Yin et al., 2021). Chen Y also confirmed that ART reduced the protein expression of p-PERK/ATF4/CHOP, and ER stress was inhibited (Chen et al., 2021). DHA reduced the phosphorylation levels of PERK, EIF2α, and IRE1α. The ER stress-mediated mitochondrial pathway was inhibited (Chen et al., 2018). The activation of CHOP was improved (Chen et al., 2019), the activation of ER stress was inhibited, and tissue damage was repaired (Li et al., 2022).

### 3.6 Lipid metabolism

The lipid metabolism disorders of podocytes are closely associated with various proteinuric kidney diseases, such as ORG, DN, MN, and MCD (Sun et al., 2021). Podocytes are susceptible to pathological lipid accumulation, leading to their dysfunction, known as lipotoxicity. Lipotoxicity is characterized by oxidative stress of mitochondrial, ERS, actin cytoskeleton remodeling, insulin resistance, and inflammatory responses, leading to hypertrophy, fusion and detachment of podocytes. There is dyslipidemia in the early stages of CKD patients, manifested by elevated levels of triglyceride (TG) and low-density lipoprotein (LDL), and low levels of high-density lipoprotein (HDL). Dyslipidemia often aggravates the deterioration of renal function (Hager et al., 2017). Significant lipid accumulation and lipid droplet formation was observed in the podocytes of patients with ORG (de Vries et al., 2014). ORG mice developed podocyte injury and proteinuria, accompanied by dysregulated lipid metabolism, ectopic lipid deposition in the kidney, the expression of the adipose differentiation-related protein, the CD36 (mediated lipid uptake and ROS release in podocytes was increased, the expression of ADRP and CD36 was significantly decreased after the inhibitor Sulfo-N-succinimidyl oleate or CD36 siRNA that blocking adipose differentiation-related protein was used, which can effectively alleviate podocyte injury (Zhao et al., 2019a). Inhibition of sterol-O-acyltransferase-1 in podocytes could block free cholesterol esterification and effectively reduce the formation of cholesterol esters and lipid droplets in human podocytes, The expression of ABC subfamily A member one and it-mediated cholesterol efflux was increased, which attenuated lipotoxicity-induced podocyte injury in DKD and Alport syndrome (Liu et al., 2020).

Artemisinin reduced the concentration of erythrocyte phospholipid, total cholesterol (TC), and LDL lipid peroxide levels and had an anti-lipogenic effect (OM., 2019). ART promoted the *ß*-oxidation of fatty acids, inhibited the synthesis of fatty acids, promoted the conversion of cholesterol to cholic acid, reduced the levels of TC, TG, and LDL, and increased HDL levels (Pu et al., 2020). Lipoprotein lipase regulates TG metabolism by hydrolyzing TG to generate free fatty acid and participates in lipid metabolism. ART upregulated lipoprotein lipase in vascular smooth muscle cells *via* KLF2/NRF2/TCF7L2 signaling pathway (He et al., 2020). ART inhibited the production of intracellular lipids in a dose-dependent manner, decreased the levels of TG and the expression of peroxisome proliferator-activated receptor-γ (PPAR-γ), fatty acid synthase (FAS), and perilipin A, which affected adipocyte differentiation (Jang, 2016). In addition, artemether reduced TG and TC levels and the expression of FAS in db/db mice, and it increased liver lipid metabolism-related enzymes, such as PPARα, acetyl-CoA carboxylase 1, and carnitine palmitoyl transferase 1 (Bai et al., 2020).

### 3.7 Glucose metabolism

The kidney is an energy-intensive organ with a very vigorous glucose metabolism. The kidneys regulate glucose homeostasis through gluconeogenesis, glucose utilization, and glucose reabsorption in the glomerular filtrate. Compared with the podocytes cultured with normal glucose levels, the protein expression of glucose-6-phosphate dehydrogenase in podocytes cultured with HG was significantly reduced, and the activated caspase-3 was increased. Further research found that HG promoted glucose-6-phosphate dehydrogenase protein degradation *via* the ubiquitin-proteasome pathway and increased podocyte apoptosis (Wang et al., 2019a), leading to an abnormal morphology and function of the podocytes, damaging the filtration barrier, leading to massive proteinuria and accelerating the progression of DN (Kang et al., 2020). HG can induce podocyte autophagy disorder and disrupt podocyte mitochondrial transmembrane potential (Kong et al., 2020).

As an artemisinin derivative, artemether exhibited potential antidiabetic activity. Artemether significantly reduced fasting blood glucose and lipid levels and improved islet function and insulin resistance in db/db mice. Artemether had positive effects on islet vacuolar degeneration and hepatic steatosis. It increased the expression of AMPK, glucose transporter 4, and insulin receptor *ß* protein in the liver of db/db mice. Further research found that artemether may regulate glucose and lipid metabolism in db/db mice by improving the immune microenvironment (Fu et al., 2020). DHA downregulated the Akt/mTOR signaling pathway, reduced the phosphorylation of Akt and mTOR, and inhibited the synthesis of glucose transporter, uptake of glucose, and the production of lactate and ATP (Zhu et al., 2018). DHA downregulated pyruvate kinase M2, a key regulator of glycolysis, inhibited the production of lactate, glucose uptake, and glycolysis (Li et al., 2019b). DHA and ART reduced the expression of glucose transporter and two key glycolysis-related enzymes, such as hexokinase and lactate dehydrogenase, as well as the levels of c-Myc in a dose and time-dependent manner. Further studies demonstrated that ART and DHA inhibited aerobic glycolysis in a c-Myc-dependent manner (Zhang et al., 2022b).

### 3.8 Insulin resistance

Insulin resistance (IR) is one of the pathological mechanisms of intrinsic cell damage in various kidney diseases. Podocytes are insulin-sensitive cells involved in stimulating insulin. They rapidly transport glucose from the tissue fluid to the cytoplasm, provide energy for the podocyte actin skeleton, and maintain the normal filtration function of podocytes. When podocytes develop IR, a large amount of glucose and lipids are deposited in the cells, resulting in podocyte injury (Xu et al., 2016). Long-term exposure to hyperglycemia or hyperinsulinemia resulted in the degradation of insulin receptor substrate and attenuated insulin signaling in podocytes (Lay et al., 2017). It promoted the down-regulation of the IRS/PI3K/Akt signaling pathway activity (Lu et al., 2021), leading to podocyte apoptosis and progression of proteinuria. Glucose uptake by podocytes is associated with elevated levels of phosphatase and tensin homolog and restoration of their homolog-mediated autophagy pathway, which can enhance glucose uptake and the expression of nephrin in the podocytes, restore IR damaged by HG and prevent HG-induced podocyte injury (Sun et al., 2017).

The intervention of artemisinin on islet function in rats with a maternal high-fat diet can reduce the size of the islets, decrease the number of *ß* cells, improve islet microcirculation, processing and sharing of insulin, reduce the ratio of proinsulin/insulin, and restore islet function by increasing the expression of PC1/3 (Chen et al., 2022). Compared with the model group, artemether reduced pancreatic *ß*-cell apoptosis, protected pancreatic *ß*-cells, and increased insulin secretion in db/db mice. When compared before treatment, artemether significantly reduced the levels of fasting blood glucose levels, increased insulin sensitivity and glucose tolerance in db/db mice, and improved considerably islet vacuolar degeneration and hepatic steatosis in db/db mice (Guo et al., 2018). Upon comparison with the untreated group, ART combined with metformin decreased the index of IR, decreased the levels of TG, TC, and LDL-C levels, and increased HDL-C levels in rats (Zhang et al., 2021a). Artemisinic acid, isolated from artemisinin, can significantly attenuate TNF-α-induced secretion of IL-6 and improve IR and the inflammatory state characterized by obesity (Lee et al., 2012).

## 4 Challenges associated with artemisinin and its derivatives upon clinical application

Since being approved by the State Food and Drug Administration, artemisinin and its derivatives have been widely used in clinics, which are not only therapeutic against malaria but also have ameliorating effects on other inflammatory immune and metabolic diseases, such as LN, DN, IgAN, renal cancer, unilateral ureteral obstruction, and cisplatin-induced kidney damage. A case reported that ART for malarial acute renal failure, which improved the loss of FP of podocyte and renal function and decreased Cr and proteinuria (Gleeson et al., 2019), and ART was safe and effective for the treatment of AKI with severely delayed hemolysis secondary to severe malaria (Leong et al., 2021).

Previous studies have shown that artemisinin and its derivatives regulate oxidative stress, show anti-inflammatory activity, demonstrate immunoregulatory effects, exhibit anti-fibrosis and anti-proliferation effects, regulate glomerular filtration, and protect kidneys (Xia et al., 2020a). Based on previous studies, this study refined the effects of artemisinin and derivatives on renal diseases, including autophagy, ER stress, glycolipid metabolism, and IR based in prior studies, and analyzes the main pathways involved in them, which laid the theoretical foundation for studying of artemisinin in renal diseases. Artemisinin and its derivatives are involved in various factors, such as TNF-α, IL-1α, IL-6, TGF-β1, ICAM-1, (Bai et al., 2019a), and signaling pathways, such as TLR4/NF-KB, NF-κB/NLRP3, and mTOR signaling pathway (Wan and Li, 2017; Wen et al., 2019; Xia et al., 2020). The occurrence and development of nephropathy are related to multiple immune inflammatory abnormalities. Artemisinin can improve oxidative stress, reduce the inflammatory response, and inhibit cellular and humoral immunity in several ways. There is a lot of experimental animal evidence that artemisinin and its derivatives have therapeutic effects on kidney diseases, but there is less literature on clinical research. Second, different researchers and groups use different effective doses of artemisinin and its derivatives for the same type of cells and animal models. Therefore, further basic experiments and clinical trials may be affected. Finally, previous studies have found that artemisinin and its derivatives are often used in the short term and in large doses to treat malaria.

In contrast, long-term and low-dose treatment regimens are primarily used in other diseases, which may induce other toxicities. For example, it further induces depolarization of the mitochondrial membrane and destroys the structure of artemisinin and its derivatives. In primary and clinical research, long-term safety testing is recommended regardless of whether the treatment time is long or short (Sun and Zhou, 2017).

## 5 Conclusion and prospects

Due to its unique characteristics, artemisinin and its derivatives have beneficial effects on kidney diseases, especially artesunate, which is soluble in weakly alkaline solution and transported by simple diffusion in the body. Artesunate exhibited high bioavailability and can be made into tablets and injections. It was found to be more suitable for preventing and treating clinical kidney diseases, making them an alternative therapy for managing kidney diseases. Moreover, they repair and delay the progression of renal pathological structure and kidney diseases by targeting pathological states, including inflammation, oxidative stress, autophagy dysfunction, mitochondrial disorder, endoplasmic reticulum stress, glycolipid metabolism disorder, and insulin resistance. More precisely, artemisinin and its derivatives can reduce the production of ROS and various inflammatory chemokines *via* different mechanisms, such as the Nrf2/HO-1 signaling pathway, NOx, TLR/NF-κB signaling pathway, and NLRP3 inflammasomes. Additionally, they can regulate podocyte autophagy, improve dysfunction caused by mitochondrial and ER stress, inhibit the synthesis of proteins that is associated with renal cell proliferation, apoptosis, and injury, regulate glucose, lipid metabolism and insulin resistance, delay and inhibit pathological stimuli, and downregulate the expression of harmful cytokines, and repair renal injury. Artemisinin and its derivatives exhibit the characteristics of multi-target, multi-pathway, and bidirectional regulation, thereby promoting the synergistic effects of each target, reducing resistance or toxic side effects of drugs, which achieve an enhanced therapeutic effect.

Increasing clinical studies have shown that artemisinin derivatives exhibited high safety and efficacy during the treatment of malaria. However, its adverse reactions cannot be ignored when used as a pharmacological intervention over a long period; thus, any discomfort in clinical patients undergoing artemisinin therapy needs to be an extra concern to clinicians.

However, most studies on kidney diseases are still in the experimental research stage based on the animal or cell models of kidney diseases, especially podocyte injury-associated kidney diseases, and more preclinical experiments and clinical trials are required to improve the exploration of mechanisms and improve the bioavailability of artemisinin and its derivatives in other systemic diseases. In conclusion, artemisinin and its derivatives are noteworthy and potentially safe and effective therapeutic drugs for treating kidney diseases.

## Glossary

**TCM**: traditional Chinese medicine
**DHA**: dihydroartemisinin
**ART**: artesunate
**DN**: diabetic nephropathy
**MN**: membranous nephropathy
**FSGS**: focal segmental glomerulosclerosis
**ORG**: obesity-related glomerulopathy
**IgN**: IgA nephropathy
**ESRD**: end-stage renal disease
**LN**: lupus nephritis
**NS**: nephrotic syndrome
**AKI**: acute kidney injury
**CKD**: chronic kidney diseases
**SM934**: β-aminoarteether maleate
**AUC**: area under the curve
**Cmax**: maximum plasma concentration
**ER**: endoplasmic reticulum
**NF-κB**: Nuclear factor-kappaB
**TNF-α**: tumour necrosis factor alpha
**IL-1β**: interleukin-1β
**RANK**: receptor activator of nuclear factor-kappaB
**HG**: high glucose
**NADPH**: Nicotinamide adenine dinucleotide phosphate
**NOX**: NADPH oxidase
**TGF-β**: transforming growth factor *ß*
**TLR4**: Toll-like receptor 4
**MyD88**: myeloid differentiation factor 88
**Bcl-2**: B cell lymphoma-2
**Bax**: Bcl2 associated X
**IFN-γ**: interferon gamma
**α-SMA**: α-smooth muscle actin
NLRP3, nucleotide leukin-rich polypeptide 3
**ASC**: apoptosis-related spot-like protein
**LPS**: lipopolysaccharide
**Ang II**: angiotensin II
**HK-2**: human kidney 2
**PAN**: puromycin aminonucleoside
**MCD**: minimal change disease
**ROS**: reactive oxygen species
**SOD**: superoxide dismutase
**Nrf2**: nuclear actor erythroid 2-related factor 2
**HO-1**: heme oxygenase-1
**Keap1**: Kelch-like ECH-associated protein-1
**ARE**: anti-oxidative response element
**MPC5**: mouse podocyte clone
**AMPK**: adenosine monophosphate-activated protein kinase
**GSK3β**: glycogen synthase kinase 3β
**STZ**: streptozotocin
**GSH-Px**: glutathione peroxidase
**MDA**: malondialdehyde
**AGEs**: advanced glycation end products
**RAGE**: receptor of advanced glycation end products
**AOPPs**: Advanced oxidation protein products
**ZO-1**: zona occludens 1
**CAT**: catalase
**UAE**: urinary albumin excretion
**eNOS**: endothelial nitric oxide synthase
**GR**: glutathione reductase
**Atg5**: autophagy-related genes 5
**LC3**: microtubule-associated protein light chain 3
**ULK**: uncoordinated 51-like kinase
**FIP200**: focal adhesion kinase family interacting protein of 200 kDa
**Vps34**: vacuole protein sorting 34/phosphatidylinositol 3-kinase
**PI3K**: phosphatidylinositol 3-kinase
**3-MA**: 3-methyladenine
**BUN**: blood urea nitrogen
**Cr**: creatinine
**AKT**: protein kinase B
**mTO**R: mammalian target of rapamycin
**PGC-1α**: Peroxisome proliferator-activated receptor γ coactivator-1α
**UPR**: unfolded protein response
**ERAD**: ER-associated protein degradation
**ATF4**: activating transcription factor 4
**eIF2α**: eukaryotic initiation factor-2α
**CHOP**: C/EBP homologous protein
**TG**: triglyceride
**TC**: total cholesterol
**LDL**: low-density lipoprotein
**HDL**: high density lipoprotein
**PPAR-γ**: peroxisome proliferator-activated receptor-γ.
